# Cases of Patients Treated in Countries With Limited Resources and Discussed by Experts of the International CML Foundation (iCMLf)—Case No. 1: A Boy Presenting With Priapism and Loss of Vision

**DOI:** 10.1155/2024/5534445

**Published:** 2024-07-29

**Authors:** Nirmalya Roy Moulik, Arlene Harriss-Buchan, Guiseppe Saglio, Nicola Evans, Meinolf Suttorp

**Affiliations:** ^1^ Tata Memorial Hospital Homi Bhabha National Institute, Mumbai, India; ^2^ International CML Foundation, Bexhill-on-Sea, UK; ^3^ Department of Clinical and Biological Sciences University of Turin, Turin, Italy; ^4^ Pediatric Hematology and Oncology Technical University of Dresden, Dresden, Germany

## Abstract

Pediatric chronic myeloid leukemia (pCML) is a rare malignancy accounting for only 2%–3% of all childhood leukemias. Due to this rarity, familiarity with pCML is limited among most pediatric practitioners, including even pediatric hemato-oncologists. In low- and middle-income countries (LMICs), limited financial resources and limited data specific to pCML represent obstacles that healthcare providers must face in diagnosing and treating this rare condition in children. The International CML Foundation (iCMLf) is improving outcomes for people with CML in these countries where resources, diagnostics, and access to medicines may be limited (https://www.cml-foundation.org/lmic-programs.html). Virtual meetings with the purpose of teaching participating pediatricians from LMICs of defined geographical regions were organised by the iCMLf in 2023. At a virtual meeting of the South Asia region, the case of a 14-year-old Indian boy was presented diagnosed with CML in a chronic phase complicated by priapism and loss of vision in his left eye due to hyperleukocytosis. Key aspects of this case are discussed in-depth from the perspective of (i) a pediatric hemato-oncologist practicing in a high-income country, (ii) a pediatric hemato-oncologist practicing in a LMIC, (iii) an adult CML hematologist, and (iv) from the iCMLf in improving the care of children with CML worldwide. Thus by discussing a multifaceted complicated case of pCML in written form as well as pointing to the pediatric module of the iCMLf Knowledge Centre will hopefully contribute to minimize existing knowledge gaps in a rare pediatric malignancy.

## 1. Introduction

The International CML Foundation (iCMLf) was established in 2009 by a group of hematologists aiming to improve the outcomes of chronic myeloid leukemia (CML) globally. Among the foundation's priorities is expanding the access to world-class CML education and best practice for physicians and scientists, no matter where in the world they are located. Integral to the work of the iCMLf is improving outcomes for patients with CML in low- and middle-income countries (LMICs) where resources, diagnostics, and access to medicines may be limited. Through an extensive online, global iCMLf communication network, international experts share their experience and advice with physicians and scientists to promote the best possible management of CML according to the available resources. Webinars, clinical preceptorships, case discussions, and educational conferences, along with tailored regional discussion groups, are facilitated by the foundation with this aim.

A key part of the LMIC program, the iCMLf Knowledge Centre chaired by G. Saglio, has evolved as a perpetual congress for the community of CML physicians [1]. Presented as an online forum by expert practitioners, the aim is to focus on decision-making in real-life clinical practice as well as in challenging or resource constraint contexts. A pediatric module of the knowledge centre chaired by N. Roy Moulik and M. Suttorp presents a comprehensive review of the treatment of pediatric CML (pCML).

Compared to adults, pCML has a 100-fold lower annual incidence being estimated between 0.06 and 0.12 cases per 100,000 children per year in high-income countries. Thus, it is a rare malignancy accounting for only 2%–3% of all childhood leukemias. Due to this rarity, familiarity with pCML is limited among most pediatric practitioners, including even pediatric hemato-oncologists. In LMICs, healthcare providers must face multiple obstacles in diagnosing and treating this rare condition in children [2].

Virtual meetings with pediatricians from LMICs of defined geographical regions (Latin America, South Asia, and Africa) were organised by the iCMLf in 2023, and pCML cases with challenging findings were presented. The authors here present one of these cases in written form, with an in-depth discussion on key aspects from the perspective of (i) a pediatric hemato-oncologist practicing in a high-income country (MS), (ii) a pediatric hemato-oncologist practicing in a LMIC (NRM), (iii) an adult CML hematologist (GS), and (iv) from the iCMLf in improving care of children with CML worldwide (AHB).

We consider this article as the first part of a series on pCML. Readers of the journal from LMICs with additional interesting cases of pCML are invited to contact the corresponding author. By sharing such case presentations, we hope to promote knowledge on pCML and point to obstacles and limitations that healthcare workers face in LMICs, and hopefully, some solutions—at least in part—are also presented.

## 2. Case Report

On October 19, 2021, a 14-year-old boy presented to a local hospital in India with complaints of intermittent fever and abdominal distension with left upper quadrant dragging sensation in the last month. The complete blood count was hemoglobin (Hb) 10.0 g/dL, white blood cell (WBC) count 430,000/*μ*L, and platelet (Plt) count 120,000/*μ*L. The peripheral blood (PB) smear differential cell count exhibited neutrophils and band cells at 38%, lymphocytes at 8%, monocytes at 2%, eosinophils at 4%, basophils at 2%, myelocytes at 20%, metamyelocytes at 20%, promyelocytes at 2%, and blasts at 4%.

A bone marrow (BM) aspiration (Oct 23, 2021) showed a hypercellular marrow with a heavily increased myeloid-to-erythroid ratio, an impressive myeloid shift to the left, with slightly increased basophils of 3% and blasts of 7%. This prompted a diagnosis of CML which was confirmed by cytogenetics applying the fluorescence in situ hybridization (FISH) technique demonstrating translocation t(9;22)(q34;q11.2)—the Philadelphia chromosome—in 98% of the interphase cells. The child was started on hydroxyurea (50 mg/kg), allopurinol (300 mg/sqm), and imatinib (300 mg/sqm) and was referred to Tata Memorial Hospital in Mumbai.

The family took about a week to reach the referral hospital, and 4 days prior to presentation in the referral hospital, the boy developed a continuous, painful penile erection, and in addition, he complained of decreased vision on his left eye for the last 2–3 days. A full blood count at the referral hospital showed Hb 9.0 g/dL, WBC 180,000/*μ*L, and Plts 810,000/*μ*L. The PB smear differential count showed neutrophils at 62%, lymphocytes at 4%, monocytes at 7%, eosinophils at 8%, basophils at 8%, myelocytes at 4%, metamyelocytes at 6%, and blasts at 1%. Karyotyping (FISH techniques) of interphase cells from PB confirmed the BCR::ABL1 gene fusion in 98% of cells, and a molecular analysis by reverse transcription-polymerase chain reaction (RT-PCR) detected the BCR::ABL1 (p210) transcript. The BM analysis confirmed the morphological findings from the referring hospital with the exception that only 1% of blasts were detected. In an immunophenotypic (FACS) analysis, no abnormal blasts were identified. A molecular analysis on tyrosine kinase domain mutations detected no variant BCR::ABL1.

Treatment was initiated with hydration paralleled with cytarabine (100 mg/m^2^) × 4 days, and hydroxyurea and imatinib were continued. This prompted a reduction of the WBC to 57,000/*μ*L by Day 5, and therefore, the leukostasis syndrome measures were stopped while hydroxyurea and imatinib were continued. By Day 10, the WBC had dropped to 12,000/*μ*L and hydroxyurea was tapered while imatinib was continued.

Approaches to treat the priapism comprised wide bore needle aspiration which did not result in penile detumescence. Therefore, on Day 2 of admission to the referral hospital (7 days from the onset of priapism), a distal shunt surgery was performed with a Bennett shunt. Ophthalmologic inspection confirmed left eye painless loss of vision. Findings concluded a vitreous hemorrhage. During the course of treatment, the boy developed secondary glaucoma which was controlled on topical antiglaucoma measures.

Results of the follow-up examinations at defined time points as indicated are listed in Table 1. Cytogenetic remission was confirmed in the PB at Month 8 and major molecular response (MMR) (MR3 = <0.1% BCR::ABL1 transcript ratio) at Month 11. With a good adherence to imatinib treatment, the child is doing well. However, the left eye has no vision and there is a total loss of penile erectile function.

## 3. Discussion

With regard to the multiple complications that the boy presented at diagnosis, the discussion is focused on the following issues:

1. Management of hyperleukocytosis in pCML.
2. What could have changed the severity of the organ damage?
3. First tyrosine kinase inhibitor (TKI) to use: imatinib vs. dasatinib/nilotinib?
4. What is an optimal follow-up strategy? (Technique: cytogenetic response vs. MR based on qRT-PCR? Material: PB vs. BM?)

### 3.1. Management of Hyperleukocytosis in pCML

What is already known about this complication? Hyperleukocytosis is defined as a WBC exceeding 100,000 leukocytes per microliter, while leukostasis describes clinical findings caused by hyperleukocytosis. Leukostasis in leukemia represents a medical emergency (pulmonary, cardiac, and cerebral failure) that needs prompt recognition and initiation of effective therapy with the intent of preserving life and preventing persistent organ failure (visual loss, hearing loss, and erectile dysfunction) [3, 4].

Hyperleukocytosis is a common finding in pCML-CP with a median WBC of 250,000 per microliter [5–7]. In a Japanese pediatric cohort, hyperleukocytosis was present at diagnosis in 162/256 (63%) pediatric patients, but treatment because of leukostasis was required in only 23 of these 162 (14%) patients. Ocular symptoms were seen in 14 cases, priapism was seen in four cases, and dyspnea, syncope, headache, knee pain, hearing difficulty, and aseptic necrosis of the femoral head were seen in one case each [8]. In the largest pediatric cohort described and analyzed for priapism to date, 16 out of 491 (3%) boys presented with this condition at diagnosis of CML [9].

The infrequent complication of leukostasis in pCML despite the median high WBC can be explained by the lower rigidity of the immature and mature cells of the myeloid compartment which form the majority of the high white cell mass. Compared to the high proportion of rigid and larger sized blasts in acute leukemias and also in CML blastic phase (CML-BP), the blasts in CML-CP usually make up less than 10% of the WBC (1%–6% in the boy discussed here).

#### 3.1.1. MS

As emphasised above, in CML-CP, the clinical signs of leukostasis require emergency treatment. Hydration is the first step and should be accompanied by fast-acting cytoreductive drugs (see Table 2). It must be remembered that the established frontline treatment by hydroxyurea and also by TKIs requires 1–2 weeks to halve the WBC, which is much too slow in an emergency scenario. Thus, leukapheresis or exchange transfusion is required as faster acting procedures.

Leukapheresis relies on the availability of sophisticated technical equipment, an apheresis team experienced in performing the procedure in children, and operational capability during emergency hours. Otherwise, leukoreduction via manual whole blood exchange transfusion (MWBET) should be planned (see below). Repeated daily treatments may be necessary until the WBC is lowered, but at pediatric age, a single procedure may relieve symptoms of leukostasis. A significant net blood volume is shifted into the leukapheresis machine, and thus, careful fluid balance to maintain circulatory euvolemia is needed [10]. As patients with pCML, like the boy discussed here, typically present at diagnosis with anemia [11], care must be taken not to worsen the red cell deficiency, and notwithstanding the risks of blood transfusion, the apheresis machine should be primed with donor blood. In addition, the caliber of venous access to ascertain the minimally required blood flow towards the separator and the risks of necessary anticoagulation in conjunction with the citrate toxicity arising from ACD-A (“Acid-Citrate-Dextrose-Adenine”) used as packed red cell stabilizer, requiring frequent calcium monitoring and supplementation, must be well balanced, especially in smaller children [12].

Factors in favor of MWBET include a low patient body weight (< 10 kg), the presence of coagulopathy including thrombocytopenia in patients with CML-BP, and the risk for other metabolic or organ dysfunctions. The low frequency of leukostasis in pediatric leukemia generally limited the MWBET experience in pediatric hemato-oncologists. Decisions should be taken based on individual experience and generally favor MWBET over leukapheresis because of smaller associated technical and complication risks [12–14].

MWBET, performed either via one central venous line (employing a push–pull technique) or using two venous cannulas for removing the patient's blood and infusing reconstituted whole blood, is usually performed to achieve an isovolumetric exchange. The recommended volumes not to be exceeded comprise an infusion/removal rate of 3 mL/kg/min for the isovolumetric exchange technique or 5 mL/kg over a 2–4-min cycle for the push–pull technique [15]. MWBET targeting at a patient's double total blood volume exchange is more effective than a single-volume exchange in leukemic cell removal. Compared to leukapheresis, which can reduce the WBC by 30%–60%, MWBET can remove up to 85% of leukocytes [16]. However, the patient's body weight and the resulting volume exchanged affect this factor [17]. Based on a bodily total blood volume of approximately 80 mL/kg, high numbers of red cell units may be required for older children [12]. Like in leukapheresis, monitoring of serum calcium is mandatory in MWBET since citrate exposure occurs with the reconstituted whole blood. Of note, in patients with CML-BP and associated thrombocytopenia, it is necessary to monitor Plts as the replacement product contains erythrocytes and fresh frozen plasma only [18].

#### 3.1.2. NRM

Our experience confirms the Japanese findings demonstrating that leukostasis-related complications are rare. In our series published recently, which is arguably the largest single centre series on pCML reported until now, we encountered a very high median WBC at presentation and over 80% of our patients had hyperleukocytosis at presentation [19]. However, very few patients (6%) had features of leukostasis. Most complications arising out of leukostasis-related organ damage are not irreversible but need prompt medical care and cytoreduction. The initial management of leukostasis is a major problem area in the resource-constrained settings. Physicians treating children in the LMICs often encounter late presentations with a potential for permanent sequelae like in this boy. The delay in presentation is primarily due to delayed care seeking by the family but also involves lack of timely appropriate care in the peripheral healthcare facilities. Lack of awareness among pediatricians and general practitioners regarding the need for time-intensive care for these situations might also be contributory.

While the rate of cytoreduction is the highest with leukapheresis or MWBET, however, the use of these invasive techniques is limited in the resource-constrained settings due to lack of easily available expertise and higher incidence of complications like bleeding and so on. In our cohort of 173 children with CML-CP, we used leukapheresis in only one child who had presented with pulmonary leukostasis needing mechanical ventilation due to hypoxia with slow response to medical cytoreduction [19]. Despite having advanced transfusion medicine services in our setting, there can be issues with timely start of leukapheresis especially during after-hours. Cytoreduction in our setting mostly comprises hyperhydration, hydroxyurea, and low-dose cytarabine along with imatinib for patients with leukostasis, whereas patients with asymptomatic hyperleukocytosis receive only hydroxyurea and imatinib. Moreover, given the delayed presentation seen in our setting and established organ damage by the time of reporting to a specialized centre, the aggressive approach required otherwise for a child with a relatively recent onset of symptoms and reversibility of organ damage may not be as rewarding. Both the boys in our series, including this boy, developed permanent loss of erectile function due to delayed intervention.

#### 3.1.3. GS

The problems related to hyperleukocytosis and its possible complications are well-known in adult patients. The main problems that I see in the LMICs are those already underlined by NRM: a high incidence of hyperleukocytosis due in part to a late referral of the pCML patients to clinical centres, and as a consequence, this can lead to a higher incidence of the possible related outcomes like in this unfortunate boy.

In my opinion, as complications due to leukostasis are observed only in a limited percentage of cases, in most of the cases, the simplest approach available also in nonspecialized centres and in LMICs is reducing the WBC starting with HU and also TKIs as soon as possible. Indeed, the clinical and hematological features of CML are very characteristic per se, and, apart from exceptionally rare forms of atypical CML, there are not so many different myeloproliferative neoplasms causing this level of WBC increase. Therefore, treatment should be started even with only a clinical and hematological diagnosis of CML or CML blast phase, because the “formal” diagnosis of a Ph-positive CML may require some days. I would also consider the possibility of adding Ara-C in cases with a WBC >250,000/*μ*L. In these cases, appropriate hydration is also recommended together with an appropriate control of a possible tumor lysis syndrome and the consequent biochemical and electrolytic alterations. In addition, for cases with even initial symptoms of leukostasis, leukapheresis (if possible) or MWBET should be undertaken as well as all the other procedures useful to resolve leukostasis complications and to lessen the risk of permanent lesions.

One of the possible initiatives able to decrease the incidence of complications of hyperleukocytosis is certainly that of increasing awareness about the urgency of intervention in these cases. This could be achieved with specific seminars and courses and could be helpful not only for pCML patients, but also in conditions like other leukemias or hematological and nonmalignant hematological diseases, for example, homozygous sickle cell anemia.

### 3.2. What Could Have Changed the Severity of the Organ Damage?

What is already known on these complications? (a) Ophthalmic problems at diagnosis of CML occur either from direct or indirect infiltration of neoplastic cells or from secondary causes like leukostasis or bleeding or a combination thereof. Although nearly all ocular structures may be affected, leukemic retinopathy is the most frequent clinical manifestation. Others include iris infiltration, anterior uveitis, hypopyon, exudative/serous retinal detachment, and optic nerve infiltration [20]. Ophthalmic conditions at diagnosis of CML are uncommon complications reported in the literature, ranging from blurred vision to severe visual loss. Retinal infiltration by leukemic cells and retinal bleeding due to leukostasis are most common in single cases or small case series, but also, more complex damage like vitreous hemorrhage, retinal vein occlusion, or optical nerve infiltration is possible in CML [20–23].

(b) Persistent, often painful, penile erection lasting more than 4 h unrelated to sexual stimulation is the definition of priapism. Classification of subtypes separates nonischemic (high flow, arterial) priapism, ischemic (low flow) priapism, and stuttering (recurrent form of ischemic priapism) priapism [24, 25]. In CML, ischemic priapism is observed in boys of all ages and not only in teenagers [8, 9, 26–28]. The erection is caused by the persistent engorgement of the corpora cavernosa due to leukostasis and reduced intracavernous blood flow resulting in hypoxia and acidosis [29, 30]

#### 3.2.1. MS

(a) Visual impairment at diagnosis of pCML is described in the form of pediatric case reports, but no systematic analysis has been performed. The boy discussed here experienced loss of left eye vision 2–3 days prior to first admission. While retinal infiltration usually resolves when CML is treated appropriately, vitreous hemorrhage and/or retinal vein occlusion, exudative retinal detachment, and/or choroidal infiltration and hemorrhage may have caused the permanent loss of vision in this boy [21–23]. Once TKI treatment has been started and normal white cell counts are achieved, surgical intervention can be planned if conservative measures do not resolve the ophthalmological complications [20].

(b) Ischemic priapism must be treated as an emergency. It can result in irreversible penile tissue damage and fibrosis if left untreated for > 48 h and consequently impotence [27, 31]. Erectile dysfunction has been reported in 35%–90% of men with priapism lasting 5–10 days [28, 32]. In parallel to rapidly working leukoreductive procedures (see above), emergency treatment comprises, in a stepwise approach, penile puncture and blood aspiration, flushing with saline, and (repeated) suprarenin injection [27, 33, 34]. In pediatric patients, these procedures should be performed under analgo-sedation. Dissociative sedation with low-dose ketamine should be used preferentially as this drug may prompt detumescence [35–38]. If priapism persists at this point, a spongio-cavernous shunt operation (like a Bennett shunt in the boy presented) is required finally.

#### 3.2.2. NRM

(a) Unilateral visual loss in this boy was caused by a massive vitreal bleed and retinal detachment due to the leukostasis and bleed. Multiple ophthalmologists in Mumbai and elsewhere in the country shared the view that the loss of vision was not correctable with conservative surgeries like vitrectomy or retinopexy.

(b) The boy under discussion presented to our hospital too late after the onset of priapism. Though a stepwise approach was followed, including a shunt surgery within 48 h of admission, the outcome unfortunately remained dismal due to the irreversible ischemic damage to the penile tissue. As mentioned earlier, both the patients with priapism in our cohort have got permanent erectile dysfunction due to delay in cytoreduction and local intervention [19].

Early diagnosis, referral, and timely management of complications are the most important interventions that are likely to limit the long-term sequelae of leukostasis in children. Raising the level of awareness among primary physicians, pediatricians, and pediatric oncologists regarding such complications seems to be the right way forward to shorten a prolonged symptom to management interval.

The reasons behind the delayed presentation of childhood cancers in LMICs can be multiple, including delayed care-seeking by the family, lack of awareness in the primary physician/pediatrician, unavailability of the needed expertise at the primary/secondary healthcare set-ups, and delayed referral to a tertiary care facility [39, 40]. As outlined in the case history, the diagnosis of CML was made before the boy developed organ damage. However, lack of adequate warning regarding the consequences of uncontrolled/unmonitored hyperleukocytosis by the referring physician, leading to the unduly long gap (1 week in this case), from referral to presentation to our centre, contributed to the dismal outcome. Transport infrastructure is unlikely to be the predominant cause for such delays, as currently, barring a few remote areas, most of the places in India are well connected to bigger cities, with centres having requisite expertise for managing children with cancer.

#### 3.2.3. GS

Very early intervention is the only way to prevent permanent organ lesions. Once there is a permanent lesion, only specific specialists in each field can evaluate and propose measures that are potentially able to reduce the damage.

### 3.3. First TKI to Use—Imatinib vs. Dasatinib/Nilotinib?

What is already known on this issue? Besides the first-generation (1G) TKI imatinib which was FDA-approved for minors in 2003, there are currently three FDA-approved second-generation (2G) TKIs (dasatinib approved in 2017, nilotinib approved in 2018, and bosutinib approved in 2023) for pediatric patients [41, 42]. When choosing which TKI to begin treatment with to obtain optimal outcomes, several factors should be considered on a case-by-case basis. These factors comprise dosing frequency (nilotinib every 12 h, all other TKIs once daily), intake with food (bosutinib), with or without food (imatinib, dasatinib) or on an empty stomach, and no food for 1 h after intake (nilotinib); the profile of side effects observed in adults as well as in children; and possible ABL1 kinase domain mutations resulting in resistance (see Table 3). Although pediatric trials generally demonstrate that pediatric patients tolerate TKIs better than adults, adolescents and young adults are known to be less compliant with regular tablet intake in chronic diseases which is not ideal when a medication may be required for many years [43–46]. Suboptimal blood drug levels may foster a greater risk of resistance and permit progression from CML-CP to advanced stages. To guide the initial treatment in adults, prognostic scoring systems and analysis of additional chromosomal aberrations (ACAs) besides the Ph chromosome are valuable tools. In pediatric patients, however, there is currently not enough data to use ACAs for risk stratification while only the EUTOS long-term survival score seems to be applicable to predict the progression-free survival [43, 47–51].

The 2G-TKIs (dasatinib and nilotinib) have been shown to induce a faster and deeper MR in adult patients in randomized trials [52, 53]. There are no randomized trials in pCML due to the small number of patients, but some published studies show similar trends [7, 54–58]. It must be pointed out that a faster and deeper MR has not been shown to impact survival. Also, data are lacking demonstrating that eligible patients for a TKI discontinuation attempt may benefit from accelerating the timeframe to achieve treatment-free remission (TFR) [43]. Nevertheless, the faster and deeper response with 2G-TKIs in adults prompted some pediatricians—mainly in the United States—to start CML treatment with a 2G-TKI because there are few serious side effects such as cardiovascular events in pediatric patients.

#### 3.3.1. MS

**Figure 1:**
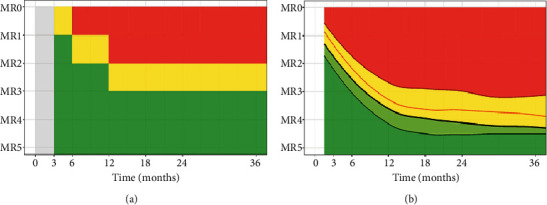
(a) Graphical illustration of the minimal residual transcript (MR) load (*y*-axis MR1 ≤ 10% BCR::ABL1, MR2 ≤ 1% BCR::ABL1, MR3 ≤ 0.1% BCR::ABL1) and interpretation of the decline over time (*x*-axis) according to the ELN criteria. The green area marks an optimal therapy response, the yellow area indicates warning, and the red area represents therapy failure. (b) Continuous references based on moving quantile analyses of optimal pediatric responders over the first 3 years of TKI therapy. The smoothed graphs illustrate the 25-quantile (green line), 50-quantile (black line), 75-quantile (orange line), and 95-quantile (red line). The light and dark green areas represent an above-average MR (optimal response), the yellow area represents a slightly below-average MR (observation or “warning” range), and the red area represents a nonoptimal MR (poor response, “failure”). The figure is depicted from Volz et al. (Sci Rep 2023; 13(1): 18199. doi:10.1038/s41598-023-45364-0) with kind permission from the authors [59].

Following leukoreduction, the recommended treatment of pCML-CP is imatinib at a dose of 260–300 mg/sqm once daily (not exceeding, in pediatric patients with a large body mass index, the adult daily dose of 400 mg) [43, 47]. Although not formally proven in children, the therapy assessment is based on the National Comprehensive Cancer Network guidelines and the European LeukemiaNet (ELN), which are derived from study data in adult CML patients [47, 48]. Besides the hematologic and cytogenetic response, in high-income countries, the focus is on the MR as measured by the BCR::ABL1 transcript level. Therapy milestones are defined by the ELN based on the transcript level at Month 3 (BCR::ABL1 ≤ 10%), Month 6 (BCR : ABL1 ≤ 1%), and Month 12 (BCR : ABL1 ≤ 0.1%), and response is categorized into optimal, warning, and failure (Figure 1(a)) [48]. Optimal response at Month 6 is a strong predictor of achieving long-term MR (MR3, ≤ 0.1% BCR::ABL1 transcript level) and also deep MR (DMR, BCR : ABL1 ≤ 0.01%) as a prerequisite for an attempt to terminate the TKI treatment after several years.

However, as children with CML typically present with much higher WBC than adults at diagnosis, the early optimal milestones are reached by approximately only 30% of children under imatinib treatment at Months 3 or 6 [7, 43, 55, 56]. Highly elevated initial WBC levels in pCML patients show a significant inverse correlation with the achievement of ELN milestones at Months 3 and 6. Therefore, instead of interpreting the MR data with sharp steps at defined timepoints, it was shown to be more appropriate in an analysis on 129 pediatric patients to interpret the MR kinetics to TKI therapy on continuous references to support an early individual therapy adjustment (Figure 1(b)) [59]. According to these graphs, the boy discussed here exhibited an optimal response with 0.028% and 0.019% BCR::ABL1 transcript ratio at Month 11 and Month 21, respectively. So, in my opinion, there is presently no need for a 2G-TKI. Most importantly in follow-up, the ELN guidelines should be adhered to for monitoring the response, identifying warning signs, and hopefully not detecting treatment failure. If no financial restrictions apply, quantitative RT-PCR monitoring should be continued in 3-month intervals.

#### 3.3.2. NRM

Our current practice is to start all newly diagnosed children with CML-CP on imatinib. The main reason for this is our long-term experience with this molecule in children, as well as the lack of cheaper alternatives as generic versions of 2G-TKIs were not available in our country until recently (generic dasatinib since 2020; generic nilotinib since 2023). We usually use the same dose as mentioned in the previous section, and the dose generally needs alteration as the children grow in height and weight. However, in patients with sustained optimum response, we tend to continue the initial dose, regardless of gain in body surface area.

We also observed that the attainment of response milestones in children is much delayed as compared to the milestones laid down in the adult guidelines. In our series, the mean time to attain MMR and DMR were 43 and 64 months, respectively [19]. Despite this delay, we have not encountered any higher incidence of events (in the form of progression to AP or BP), so much so that the long-term EFS of our cohort is over 95%.

Both generic and brand imatinib are available in our country. The Glivec International Patient Assistance Program (GIPAP) has stopped enrolling new patients since 2016, and generic imatinib, being much cheaper, is currently used by most centres in our country as the first-line agent for pCML. While the data on bioavailability and other pharmacokinetic indicators of the generic preparations are limited, our retrospective analysis shows that the time taken to attain MMR and DMR in our cohort was significantly lesser in children on generic formulation [19]. While this analysis was not planned and does not provide a head-to-head comparison as in a prospective randomized study, the findings can, at the least, be reassuring to physicians from the LMIC regions where access to brand imatinib is limited by unavailability and cost.

The higher price of 2G-TKIs may have a big impact on the decision about which TKI to select for upfront treatment, especially in LMICs. In India, the healthcare system is heterogenous ranging from government-funded hospitals/teaching hospitals and institutes to corporate hospitals, leading to lack of equity in access to healthcare [60]. While some of the government health schemes cover expenditure (with a cap) for diagnosis and treatment of common cancers including CML, the more affluent section of the population has their healthcare needs covered by private insurance providers or by self-funding. The hospital where this patient is being treated is a government-aided hospital, and treatment provided to children with cancer is completely free of cost to those who cannot afford it. As of note, several reports have demonstrated that imatinib provides a more cost-effective treatment approach than dasatinib or nilotinib for patients who were first diagnosed with CML-CP, mostly owing to the availability of generics [61–63].

Though TFR is likely to benefit children with CML more than adults and starting a 2G-TKI can make patients eligible for TFR earlier, at the moment, we are sticking to imatinib as the first-line treatment until more data is available. The 2G-TKIs are still reserved for patients with imatinib intolerance/toxicity or treatment failure.

#### 3.3.3. GS

This is a very important point: why is the response to imatinib delayed in pCML with respect to adults and why is generic imatinib (that was introduced later than the brand drug), apparently better in inducing faster responses? Is this due to problems of initial compliance in giving the correct dose to children due to concerns about side effects or to differences in the absorption, in the intracellular transport, or in the metabolism of imatinib in children? At the moment, we do not know the answers to these questions, and they should be further investigated in order to provide insights into the most effective TKI to be used as first-line therapy and to shorten the achievement of DMR to try to discontinue the TKI therapy [64].

As most of the 2G-TKIs are now becoming generic and their price is expected to become affordable even in LMICs, I think that we need clinical studies to verify possible advantages of the use of 2G-TKIs as first-line therapies in pCML, even at lower dosages with respect to those recommended in registration studies as demonstrated recently in adults [65, 66]. Although we do not expect advantages in terms of overall survival (OS), we can expect that a higher percentage of patients could reach DMR and the parameters recommended to try to discontinue the TKI therapy in a shorter period of time. TFR should now become the ideal primary goal for TKI therapy in pCML. Because of their long-life expectancy, young pCML patients should face the possibility of real “cure” and to avoid, as much as possible, toxicity related to TKI therapy during their development and growth, and long-term toxicity due to lifelong TKI therapy.

### 3.4. What Test and What Material Is the Basis to Perform an Optimal Follow-Up Strategy?

#### 3.4.1. How Does Karyotyping (Cytogenetic Response) Compare to MR (Based on qRT-PCR)? What Material Should Be Investigated (PB vs. BM)?

Monitoring of MR using PCR for BCR::ABL1 is a pivotal tool for guiding TKI therapy in the long-term follow-up of patients with CML. Specific time-dependent milestones for definition of grade of response and treatment failure (see Section 3.3.1) have been included in therapy recommendations for adults and children [47, 48]. Optimal monitoring should make use of qRT-PCR if this sophisticated and expensive technique is available and affordable, and standardized results should be interpreted according to the international scale (IS) [67]. Analysis is optimally performed monthly during the first 3–4 months after starting TKI treatment to identify the slope of the hopefully declining BCR::ABL1 transcript ratio and thereafter in 3-month intervals [68].

Cytogenetic analysis by chromosomal banding technique of marrow cell metaphases is mandatory at diagnosis to detect ACA besides the Ph chromosome. It may also be useful when performed during TKI therapy, but alone is not sufficiently sensitive for response monitoring. It must be remembered that a BCR::ABL1 transcript ratio of approximately 1% (MR2) corresponds with a complete cytogenetic response (CCyR) (no detectable Ph chromosome). However, in patients with atypical chromosomal translocations, atypical BCR::ABL1 transcripts that cannot be measured by standard qRT-PCR, and treatment failure/resistance, karyotyping should be performed. Also, FISH monitoring may be useful in patients with atypical transcripts.

#### 3.4.2. MS

**Figure 2:**
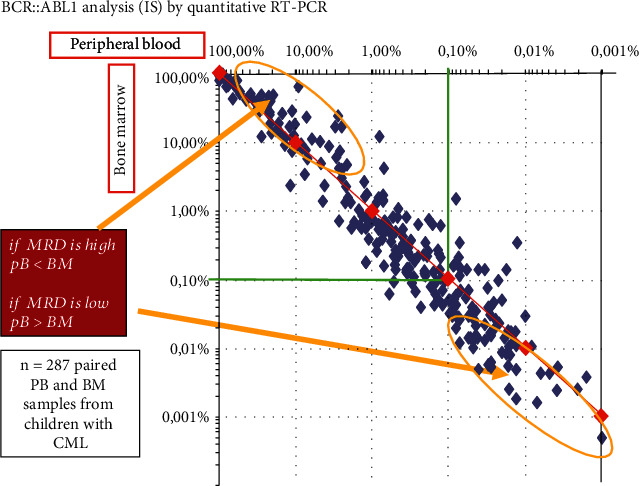
Correlation of BCR::ABL1 transcript rate (IS) in a specimen from peripheral blood and bone marrow collected in parallel from children with CML (M. Suttorp and C. Thiede, unpublished results). Abbreviations: BM, bone marrow; MRD, measurable residual disease; PB, peripheral blood.

Also in pCML, it has become common practice to use PB instead of BM aspirate to monitor residual disease [47]. When BCR::ABL1 transcript levels are investigated by qRT-PCR in paired PB and BM specimens, a good overall concordance of BCR::ABL1 (IS) results is generally found, thus supporting the current practice to primarily use PB for long-term molecular follow-up monitoring in CML. Specimens from CML patients in major MR (MR3, BCR::ABL1 transcript rate ≤ 0.1%) show a systematic tendency towards higher levels in PB [69]. This difference may result in a lower rate of DMR when BCR::ABL1 (IS) is assessed in the PB compared to BM aspirates (see Figure 2). Thus, the classification of DMR is more stringent in PB than in BM.

#### 3.4.3. NRM

Despite availability of both FISH and qRT-PCR, we tend to monitor the response by 3 monthly FISH until CCyR is attained. Thereafter, qRT-PCR is done every 3–6 months until the attainment of MMR. Once MMR is attained, further monitoring is done using qRT-PCR every 6–12 months. The reason for using FISH instead of qRT-PCR is mainly due to the fact that the latter is three to four times costlier than the former.

In deviation from the usual recommendation of monitoring qRT-PCR every 3 months indefinitely, we start doing them at a lesser frequency (every 6–12 months) after MMR is attained which is mostly due to financial reasons. Because the goal of therapy as of now is MMR, the incremental value of more frequent monitoring after attainment of MMR in our setting is difficult to justify. Though there is a theoretical risk of missing a loss in response sufficiently early, this approach has not posed any significant clinical problem in our cohort yet.

#### 3.4.4. GS

I think that the schedule suggested by NRM to use FISH on PB until the achievement of negativity (limit of sensitivity approximately 0.5% in our hands) followed by RQ-PCR every 3–6 months until the confirmed achievement of MMR, then switching to a frequency of at least one per year, although not optimal, could be the most realistic and acceptable monitoring strategy in LMICs. Of course, if possible, the classical and recommended RQ-PCR analysis every 3–4 months starting after the 3rd month from the beginning of the TKI therapy would be the optimal schedule to monitor the response and to drive more rapid changes of therapy due to onset of resistance. But we do not know if this could produce substantial benefits in terms of OS with respect to a slightly later reaction. Comparative studies are probably needed.

### 3.5. AHB: Role of the iCMLf in Improved Outcomes for pCML Patients Around the World

Numerous facets of this case underscore the imperative for the iCMLf's endeavours in supporting and educating physicians in LMICs.

a. The rarity of CML in children presents a formidable challenge, as many pediatricians and primary care physicians lack direct experience in its management, compounded by a dearth of peers with relevant expertise. The foundation's global networking capabilities play a pivotal role in bridging this knowledge gap, connecting physicians with enquiries to CML experts who can provide prompt advice. This can be done directly on request for assistance or using the foundation's online “Case Discussion Forum” [70]. A possible solution might be organizing specific units/group, taking care of all CML patients, both adult and pediatric, in reference hospitals, similar to what has already been done for thalassemia and hemoglobinopathy patients. This could also achieve uniform, high-quality assistance in terms of appropriate treatments, not only for CML, but also for all its complications and side effects that can arise during treatment [71]. Providing appropriate monitoring should become more convenient, and in general, it would provide a better balance between effort, cost, and quality of care.
b. Delayed access to appropriate care is commonly reported throughout LMICs. As mentioned previously by NRM, the reasons behind delayed presentation can be multiple and patients with CML are often initially treated for conditions other than CML, for example, malaria or tuberculosis. The iCMLf faces the ongoing challenge of expanding awareness of CML diagnosis and treatment protocols outside the major treatment facilities in LMICs. The foundation also works to expand the use of its educational resources across the entire spectrum of physicians and scientists managing CML, extending beyond those with a specific focus on the disease.
c. Expense and access to diagnostics and treatment. For most people with CML, management requires adherence to lifelong TKI therapy along with frequent disease monitoring [47]. By initiating programs aimed at facilitating access to appropriate diagnostic tools, and promoting sustainable healthcare infrastructures, the iCMLf endeavours to improve CML diagnosis and management in underserved areas. This coupled with the knowledge of appropriate therapy management maximises the use of available TKIs and improves patient outcomes.
d. Challenges managing uncommon complications of CML. In the context of the presented case, the adverse outcomes related to priapism and ophthalmological features might have been avoided if initial caregivers had been cognisant of potential complications, forewarned the family, and emphasised the urgency of presenting to the referral centre. The pCML module of the iCMLf Knowledge Centre [1] is a comprehensive repository on CML treatment in children. This is supplemented by additional knowledge centre topics, such as insights on ophthalmologic manifestations and the medical and surgical management of priapism.

This case highlights gaps in capacity and challenges arising from resource limitations in LMICs. Drawing insights from this and other complex cases, the authors propose the development of context-specific recommendations for treating pCML specifically addressing the challenges faced in LMICs. The iCMLf, given its strategic positioning, is well-suited to explore and coordinate the development of these recommendations, ultimately contributing to improved outcomes for children with CML.

## Figures and Tables

**Table 1:** Hematological, cytogenetic, and molecular findings during the course of treatment with imatinib.

**Date time since Dx**	**Nov 2021** **Month 1**	**Feb 2022** **Month 4**	**June 2022** **Month 8**	**Sept 2022** **Month 11**	**July 2023** **Month 21**
Hb (g/dL)	9.0	12.8	12.0	12.8	12.8
White cell count (cells/*μ*L)	180,000	8800	6600	6100	7100
Platelet count (cell/*μ*L)	81,000	106,000	141,000	139,000	146,000
Cytogenetics (FISH) BCR::ABL1 fusion	98% (BM)	10% (PB)	Negative (PB)	n.d.	n.d.
Quant. RT-PCR BCR::ABL1 p210 transcript	n.d.	n.d.	n.d.	0.028 (PB)	0.019 (PB)

Abbreviations: BM, bone marrow; Dx, diagnosis; FISH, fluorescent in situ hybridization; n.d., not done; PB, peripheral blood.

**Table 2:** Speed of leukoreduction depending on the selected treatment.

**Drug/measure**	**Time for halving the WBC**
Hydroxyurea (50 mg/kg)	1–2 weeks
TKI	1–2 weeks
Low dose Ara-C (100 mg iv 24 h)	3–5 days
Low dose Ara-C plus thioguanine (1 mg/kg, max. 40 mg once daily)	3 days
Leukapheresis/exchange transfusion	30%–50%/80% reduction within hours

**Table 3:** Major side effects and resistance profile of TKIs licensed for minors with CML.

**TKI**	**Frequently observed side effects**	**Resistance against mutation**
Imatinib	Edema, diarrhea, longitudinal growth retardation, musculoskeletal pain	Multiple mutations
Dasatinib	Longitudinal growth retardation, pleural/pericardial effusions, pulmonary hypertension, gastrointestinal bleeding, QTc prolongation	V299L, T315I/A, F317L/V/I/C
Nilotinib	Longitudinal growth retardation, QTc prolongation, metabolic changes, (glucose/lipids), arterial occlusion	Y253H, E255K/V, T315I, F359V/C/I
Bosutinib	(No data yet on longitudinal growth retardation), diarrhea, hepatic enzyme increase	V299L, G250E, T315I, F317L

## Data Availability

Data with the limitation that the identity of the patient cannot be disclosed are available on written request from the first author, Prof. Dr. Nirmalya Roy Moulik, Pediatric Oncology, Tata Memorial Hospital, Homi Bhabha National Institute, Dr. E. Borges Road, Mumbai 400094, India (e-mail: roymoulik@gmail.com).

## References

[1] https://knowledge.cml-foundation.org.

[2] Roy Moulik N., Harriss-Buchan A., Saglio G., Suttorp M. (2024). Challenges in management of pediatric chronic myeloid leukemia (pCML) in the low-middle income countries (LMICs): insights from an International CML Foundation (iCMLf) Multi-National Survey. *Pediatric Hematology and Oncology*.

[3] Röllig C., Ehninger G. (2015). How I treat hyperleukocytosis in acute myeloid leukemia. *Blood*.

[4] Macaron W., Sargsyan Z., Short N. J. (2022). Hyperleukocytosis and leukostasis in acute and chronic leukemias. *Leukemia & Lymphoma*.

[5] Millot F., Traore P., Guilhot J. (2005). Clinical and biological features at diagnosis in 40 children with chronic myeloid leukemia. *Pediatrics*.

[6] Dou X., Zheng F., Zhang L. (2021). Adolescents experienced more treatment failure than children with chronic myeloid leukemia receiving imatinib as frontline therapy: a retrospective multicenter study. *Annals of Hematology*.

[7] Suttorp M., Schulze P., Glauche I. (2018). Front-line imatinib treatment in children and adolescents with chronic myeloid leukemia: results from a phase III trial. *Leukemia*.

[8] Kurosawa H., Tanizawa A., Tono C. (2016). Leukostasis in children and adolescents with chronic myeloid leukemia: Japanese Pediatric Leukemia/Lymphoma Study Group. *Pediatric Blood & Cancer*.

[9] Suttorp M., Sembill S., Kalwak K., Metzler M., Millot F. (2023). Priapism at diagnosis of pediatric chronic myeloid leukemia: data derived from a large cohort of children and teenagers and a narrative review on priapism management. *Journal of Clinical Medicine*.

[10] McLeod B., McLeod B. (2004). Pediatric therapeutic apheresis. *Therapeutic Apheresis: A Physician’s Handbook*.

[11] Delehaye F., Rouger J., Brossier D. (2023). Prevalence of anemia at diagnosis of pediatric chronic myeloid leukemia and prognostic impact on the disease course. *Annals of Hematology*.

[12] Runco D. V., Josephson C. D., Raikar S. S. (2018). Hyperleukocytosis in infant acute leukemia: a role for manual exchange transfusion for leukoreduction. *Transfusion*.

[13] Creutzig U., Rössig C., Dworzak M. (2016). Exchange transfusion and leukapheresis in pediatric patients with AML with high risk of early death by bleeding and leukostasis. *Pediatric Blood & Cancer*.

[14] Oberoi S., Lehrnbecher T., Phillips B. (2014). Leukapheresis and low-dose chemotherapy do not reduce early mortality in acute myeloid leukemia hyperleukocytosis: a systematic review and meta-analysis. *Leukemia Research*.

[15] Ramasethu J., Mac Donald M. R., Jayashree R. (2013). Exchange transfusion. *Atlas of procedures in neonatology*.

[16] Schwartz J., Padmanabhan A., Aqui N. (2016). Guidelines on the use of therapeutic apheresis in clinical practice–evidence‐based approach from the writing committee of the American Society for Apheresis: the seventh special issue. *Journal of Clinical Apheresis*.

[17] Jain R., Bansal D., Marwaha R. K. (2013). Hyperleukocytosis: emergency management. *Indian Journal of Pediatrics*.

[18] Bunin N. J., Kunkel K., Callihan T. R. (1987). Cytoreductive procedures in the early management in cases of leukemia and hyperleukocytosis in children. *Medical and Pediatric Oncology*.

[19] Roy Moulik N., Keerthivasagam S., Pandey A. (2024). Treatment and follow-up of children with chronic myeloid leukaemia in chronic phase (CML-CP) in the tyrosine kinase inhibitor (TKI) era-two decades of experience from the Tata Memorial Hospital paediatric CML (pCML) cohort. *British Journal of Haematology*.

[20] Yassin M. A., Ata F., Mohamed S. F. (2022). Ophthalmologic manifestations as the initial presentation of chronic myeloid leukemia: a review. *Survey of Ophthalmology*.

[21] Almater A. I., Alhadlaq G. S., Alromaih A. Z. (2022). Unilateral Subhyaloid Hemorrhage as a Presenting Sign of Chronic Myeloid Leukemia. *American Journal of Case Reports*.

[22] Hsia N. Y., Lin C. J., Lin H. J., Wu K. H. (2020). Foveal photoreceptors loss and then recovery after treatment in a chronic myelogenous leukemia patient. *Pediatric Hematology and Oncology*.

[23] Goel N., Pangtey B., Thakar M., Raina U. K., Ghosh B. (2012). Chronic myeloid leukemia presenting with bilateral central retinal vein occlusion and massive retinal infiltrates. *Journal of AAPOS*.

[24] Ericson C., Baird B., Broderick G. A. (2021). Management of priapism. *Urologic Clinics of North America*.

[25] Salonia A., Eardley I., Giuliano F. (2014). European Association of Urology guidelines on priapism. *European Urology*.

[26] Jesus L. E., Dekermacher S. (2009). Priapism in children: review of pathophysiology and treatment. *The Journal of Pediatrics*.

[27] Donaldson J. F., Rees R. W., Steinbrecher H. A. (2014). Priapism in children: a comprehensive review and clinical guideline. *Journal of Pediatric Urology*.

[28] Ali E., Soliman A., De Sanctis V., Nussbaumer D., Yassin M. (2021). Priapism in patients with chronic myeloid leukemia (CML): a systematic review. *Acta Bio-Medica*.

[29] Rodgers R., Latif Z., Copland M. (2012). How I manage priapism in chronic myeloid leukaemia patients. *British Journal of Haematology*.

[30] Broderick G. A., Kadioglu A., Bivalacqua T. J., Ghanem H., Nehra A., Shamloul R. (2010). Priapism: pathogenesis, epidemiology, and management. *The Journal of Sexual Medicine*.

[31] Dekalo S., Stern N., Broderick G. A., Brock G. (2022). Priapism or prolonged erection: is 4–6 hours of cavernous ischemia the time point of irreversible tissue injury?. *Sexual Medicine Reviews*.

[32] Morano S. G., Latagliata R., Carmosino I., Girmenia C., Dal Forno S., Alimena G. (2000). Treatment of long-lasting priapism in chronic myeloid leukemia at onset. *Annals of Hematology*.

[33] Vilke G. M., Harrigan R. A., Ufberg J. W., Chan T. C. (2004). Emergency evaluation and treatment of priapism. *The Journal of Emergency Medicine*.

[34] van der Velde M. G. A. M., Tiellemans S. M. B., de Lil H., Nieuwenhuizen L. (2022). The value of leukapheresis for treatment of priapism as presenting feature of chronic myeloid leukemia - case report and review of literature. *EJHaem*.

[35] Bergman S. A. (1999). Ketamine: review of its pharmacology and its use in pediatric anesthesia. *Anesthesia Progress*.

[36] Benzon H. T., Leventhal J. B., Ovassapian A. (1983). Ketamine treatment of penile erection in the operating room. *Anesthesia and Analgesia*.

[37] Villalonga A., Beltran J., Gomar C., Nalda M. A. (1985). Ketamine for treatment of priapism. *Anesthesia and Analgesia*.

[38] Zipper R., Younger A., Tipton T. (2018). Ischemic priapism in pediatric patients: spontaneous detumescence with ketamine sedation. *Journal of Pediatric Urology*.

[39] Faruqui N., Bernays S., Martiniuk A. (2020). Access to care for childhood cancers in India: perspectives of health care providers and the implications for universal health coverage. *BMC Public Health*.

[40] Nath A., Mathur P., Sudarshan K. L. (2023). An assessment of childhood cancer care services in India - gaps, challenges and the way forward. *Lancet Reg Health Southeast Asia*.

[41] Phillips L. N., Hijiya N. (2021). Tyrosine kinase inhibitors and beyond for chronic myeloid leukemia in children. *Paediatric Drugs*.

[42] Hoy S. M. (2024). Bosutinib: pediatric first approval. *Paediatric Drugs*.

[43] Hijiya N., Suttorp M. (2019). How I treat chronic myeloid leukemia in children and adolescents. *Blood*.

[44] Millot F., Claviez A., Leverger G., Corbaciglu S., Groll A. H., Suttorp M. (2014). Imatinib cessation in children and adolescents with chronic myeloid leukemia in chronic phase. *Pediatric Blood & Cancer*.

[45] Hijiya N., Millot F., Suttorp M. (2015). Chronic myeloid leukemia in children. *Pediatric Clinics of North America*.

[46] Hijiya N., Schultz K. R., Metzler M., Millot F., Suttorp M. (2016). Pediatric chronic myeloid leukemia is a unique disease that requires a different approach. *Blood*.

[47] Athale U., Hijiya N., Patterson B. C. (2019). Management of chronic myeloid leukemia in children and adolescents: recommendations from the Children's Oncology Group CML Working Group. *Pediatric Blood & Cancer*.

[48] Hochhaus A., Baccarani M., Silver R. T. (2020). European LeukemiaNet 2020 recommendations for treating chronic myeloid leukemia. *Leukemia*.

[49] Millot F., Guilhot J., Suttorp M. (2017). Prognostic discrimination based on the EUTOS long-term survival score within the International Registry for Chronic Myeloid leukemia in children and adolescents. *Haematologica*.

[50] Millot F., Dupraz C., Guilhot J. (2017). Additional cytogenetic abnormalities and variant t(9;22) at the diagnosis of childhood chronic myeloid leukemia: the experience of the International Registry for Chronic Myeloid Leukemia in children and adolescents. *Cancer*.

[51] Karow A., Göhring G., Sembill S. (2022). The cytogenetic landscape of pediatric chronic myeloid leukemia diagnosed in chronic phase. *Cancers (Basel)*.

[52] Hochhaus A., Saglio G., Hughes T. P. (2016). Long-term benefits and risks of frontline nilotinib vs imatinib for chronic myeloid leukemia in chronic phase: 5-year update of the randomized ENESTnd trial. *Leukemia*.

[53] Cortes J. E., Saglio G., Kantarjian H. M. (2016). Final 5-year study results of DASISION: the dasatinib versus imatinib study in treatment-naïve chronic myeloid leukemia patients trial. *Journal of Clinical Oncology*.

[54] Millot F., Baruchel A., Guilhot J. (2011). Imatinib is effective in children with previously untreated chronic myelogenous leukemia in early chronic phase: results of the French national phase IV trial. *Journal of Clinical Oncology*.

[55] Millot F., Guilhot J., Baruchel A. (2014). Impact of early molecular response in children with chronic myeloid leukemia treated in the French Glivec phase 4 study. *Blood*.

[56] Giona F., Saglio G., Santopietro M. (2018). Early response does not predict outcome in children and adolescents with chronic myeloid leukaemia treated with high-dose imatinib. *British Journal of Haematology*.

[57] Hijiya N., Maschan A., Rizzari C. (2019). Phase 2 study of nilotinib in pediatric patients with Philadelphia chromosome-positive chronic myeloid leukemia. *Blood*.

[58] Gore L., Kearns P. R., de Martino M. L. (2018). Dasatinib in pediatric patients with chronic myeloid leukemia in chronic phase: results from a phase ii trial. *Journal of Clinical Oncology*.

[59] Volz C., Zerjatke T., Gottschalk A. (2023). Continuous therapy response references for BCR::ABL1 monitoring in pediatric chronic myeloid leukemia. *Scientific Reports*.

[60] Kumar A. (2023). The transformation of the Indian healthcare system. *Cureus*.

[61] Li N., Zheng B., Cai H. F. (2018). Cost effectiveness of imatinib, dasatinib, and nilotinib as first-line treatment for chronic-phase chronic myeloid leukemia in China. *Clinical Drug Investigation*.

[62] Nguyen J. T., Cole A. L., Leech A. A., Wood W. A., Dusetzina S. B. (2020). Cost-effectiveness of first-line tyrosine kinase inhibitor therapy initiation strategies for chronic myeloid leukemia. *Value in Health*.

[63] Agrawal R., Vieira J., Ryan J. (2022). A systematic literature review of the economic evaluations of treatments for patients with chronic myeloid leukemia. *PharmacoEconomics*.

[64] Tiribelli M., Latagliata R., Breccia M. (2023). Determinants of frontline tyrosine kinase inhibitor choice for patients with chronic-phase chronic myeloid leukemia: a study from the Registro Italiano LMC and Campus CML. *Cancer*.

[65] Cortes J., Rea D., Lipton J. H. (2019). Treatment-free remission with first- and second-generation tyrosine kinase inhibitors. *American Journal of Hematology*.

[66] Claudiani S., Apperley J. F., Szydlo R. (2021). TKI dose reduction can effectively maintain major molecular remission in patients with chronic myeloid leukaemia. *British Journal of Haematology*.

[67] Cross N. C., White H. E., Colomer D. (2015). Laboratory recommendations for scoring deep molecular responses following treatment for chronic myeloid leukemia. *Leukemia*.

[68] Branford S., Apperley J. F. (2022). Measurable residual disease in chronic myeloid leukemia. *Haematologica*.

[69] Greiner G., Ratzinger F., Gurbisz M. (2020). Comparison of BCR-ABL1 quantification in peripheral blood and bone marrow using an international scale-standardized assay for assessment of deep molecular response in chronic myeloid leukemia. *Clinical Chemistry and Laboratory Medicine*.

[70] http://www.cml-foundation.org/icmlf-forum.html.

[71] Malhotra H., Radich J., Garcia-Gonzalez P. (2019). Meeting the needs of CML patients in resource-poor countries. *Hematology 2014, the American Society of Hematology Education Program Book*.

